# Neighborhood Characteristics at Birth and Positive and Negative Psychotic Symptoms in Adolescence: Findings From the ALSPAC Birth Cohort

**DOI:** 10.1093/schbul/sbz049

**Published:** 2019-06-05

**Authors:** Francesca Solmi, Glyn Lewis, Stanley Zammit, James B Kirkbride

**Affiliations:** 1 Division of Psychiatry, UCL, London, UK; 2 Division of Psychological Medicine and Clinical Neurosciences, MRC Centre for Neuropsychiatric Genetics and Genomics, Cardiff University, Cardiff, UK; 3 Centre for Mental Health, Addiction and Suicide Research, School of Social and Community Medicine, University of Bristol, Bristol, UK

**Keywords:** psychotic experiences, negative symptoms, neighborhood, cohort study, ALSPAC, polygenic risk scores

## Abstract

**Background:**

Urban birth is associated with risk of non-affective psychoses, but the association with subclinical positive and negative symptoms is less clear, despite emerging evidence. Further the extent to which these findings are confounded by polygenic risk scores (PRS) for schizophrenia is also unknown.

**Methods:**

Using data from the Avon Longitudinal Study of Parents and Children, linked to census geographical indicators, we examined whether various indices of urbanicity at birth were associated with negative and positive psychotic symptoms at age 16 and 18 years, respectively. We used logistic regression models, controlling for child’s ethnicity, maternal age, education, marital status, social class, depressive symptoms, other neighborhood exposures, and, in a subsample of children of white ethnicity (*N* = 10 283), PRS for schizophrenia.

**Results:**

Amongst 11 879 adolescents, those born in the most densely populated tertile had greater odds of reporting positive psychotic experiences, after multivariable adjustment (odds ratio [OR]: 1.57, 95% confidence intervals (CIs): 1.14–2.17). Adolescents born in the most socially fragmented neighborhoods had greater odds of negative symptoms, after multivariable adjustment (OR: 1.43, 95% CI: 1.06–1.85). Although we found that greater schizophrenia PRS were associated with an increased risk of being born in more deprived and fragmented (bot not more densely populated areas), these associations were not confounded by PRS.

**Interpretation:**

Birth into more densely populated and socially fragmented environments increased risk of positive and negative psychotic phenomena in adolescence, respectively, suggesting that different forms of neighborhood social adversity may impinge on different psychopathophysiologies associated with the clinical expression of psychosis.

## Introduction

Psychotic disorders may lie at one end of an “extended psychosis phenotype,”^1,2^ which includes transient symptoms and subthreshold psychotic experiences that may, for some, be a precursor for later clinical diagnoses. Accordingly, epidemiological and clinical research show that psychotic experiences share etiological overlap with clinical disorders, including neurodevelopmental,^3–6^ pre- and perinatal,^7,8^ and environmental^9–14^ exposures. For example, schizophrenia is more common among individuals born in more densely populated and deprived areas,^15–18^ exhibiting a dose–response relationship,^16^ and some, although not all,^19^ emerging evidence suggests that subthreshold psychotic experiences follow similar patterns with respect to urban birth^9–11^ and residence.^12–14^ However, no study to date has investigated whether specific aspects of the social environment at birth (ie deprivation, social fragmentation, population density) are related to subclinical psychotic symptoms. Further, most studies of subclinical symptoms have focused on positive psychotic phenomena, with only 2 studies having investigated the effect of urban living on negative symptoms, finding equivocal results.^20,21^ Studying whether the urbanicity–psychosis association is general in nature, or specific to certain symptoms could provide important insights on causal mechanisms underlying etiology.^20^

To enhance our causal understanding, 4 recent studies have investigated whether observed associations between neighborhood characteristics and schizophrenia are the results of genetic predisposition.^22–25^ For example, a recent Danish study found that greater polygenic risk scores (PRS) for schizophrenia were associated with increased odds of living in urban environment at age 15 years,^22^ in line with 2 other studies.^23,25^However, only 1 study has considered whether PRS for schizophrenia is associated with urban birth, finding no association with settlement size in Denmark.^22^ No study has yet examined whether PRS for schizophrenia is associated with a broader set of social environmental risk factors for psychosis,^10,26^,^27^including neighborhood deprivation, social fragmentation, population density, and inequality at birth.

Two studies which have directly examining whether genetic factors confounded the association between neighborhood characteristics and psychosis have yielded equivocal results. One register-based family study from Sweden found that the association between neighborhood deprivation and population density and schizophrenia disappeared in analyses restricted to sibling pairs discordant for neighborhood exposures at age 15 years.^28^ Nevertheless, a second study found that controlling for PRS did not confound the association between urbanicity and schizophrenia.^22^ To the best of our knowledge, no study has tested whether schizophrenia PRS confound the association between neighborhood characteristics at birth and positive and negative psychotic symptoms in the general population.

Using genetically and environmentally well-characterized longitudinal data from a population-based birth cohort, we therefore investigated (1) whether markers of the social environment at birth, including population density, deprivation, inequality, and social fragmentation were associated with positive and negative psychotic symptoms in adolescence; (2) whether PRS for schizophrenia were associated with social environments at birth; and (3) whether PRS for schizophrenia potentially confounded any associations between social environments at birth and positive or negative psychotic symptoms in adolescence. Consistent with available evidence,^21,29^ we hypothesized that positive symptoms would be associated with greater population density at birth, and that deprivation and social fragmentation would be associated with both positive and negative symptoms.

## Methods

### Sample

The Avon Longitudinal Study of Parents and Children (ALSPAC) is an ongoing longitudinal birth cohort that recruited pregnant women living in the former region of Avon (Bristol, United Kingdom) with an expected due date between April 1, 1991 and December 31, 1992 (*n* = 14 541).^30^ Here, we included children in the core ALSPAC sample (*n* = 14 062) who were alive at 1 year of age (*n* = 13 998), whose mothers’ addresses in pregnancy could be linked to census geographical indicators (ie complete exposure data; supplementary figure S1). Where applicable, only the first-born twin was included to control for shared genetic and environmental effects. All mothers gave informed consent to participate in the study. The ALSPAC ethics and law committee and local research ethics committees approved this study. More details on the ALSPAC cohort can be found at: www.bristol.ac.uk/alspac, which also contains a fully searchable data dictionary available at: http://www.bris.ac.uk/alspac/researchers/data-access/data-dictionary/.

## Measures

### Neighborhood Social Environments at Birth

At 32-week gestation, residential addresses of 11 013 mothers were linked to enumeration district (ED) indicators, a small-area administrative division used in the 1991 census (median population: 518 people; interquartile range: 446–593). For 866 mothers missing postal information at 32 weeks, we used their address at the 8-week assessment, given high correlation between addresses available at both assessments (*r* = .98). For each participant, we derived measures of neighborhood-level population density, deprivation, inequality, and fragmentation from 1991 Census data at ED-level (supplementary material for detailed descriptions). We divided these continuous measures into tertiles of equal numbers of participants. Supplementary table S1 shows the distribution of neighborhood-level exposures in the ALSPAC sample, by tertile, relative to England.

### Negative Symptoms

Negative symptoms were measured by postal questionnaire at age 16.5 years using 10 items from the Community Assessment of Psychic Experiences, scored on a 4-point Likert scale (0 = never, 1 = sometimes, 2 = often, 3 = always). Questions sought to assess the presence of behaviors such as apathy (“Have you felt that you are spending all your days doing nothing?”) or lack of interest in social relationships (“Have you felt that you have no interest to be with other people?”). We derived a total score by summing these items, dichotomized using a cut-off of 14, as in previous publications.^31^

### Psychotic Experiences

Psychotic experiences were measured at clinical assessment at age 18 years using the Psychotic-Like Symptoms Interview, a semi-structured interview assessing the presence of delusions, hallucinations, and intrusive thoughts, using items derived from the Diagnostic Interview Schedule for Children version IV^32^ and the Schedule for Clinical Assessment in Neuropsychiatry.^33^ Consistent with several previous ALSPAC studies^31,34–36^ we used a binary measure to denote either absence, or presence of suspected or definite psychotic experiences.

### Polygenic Risk Scores for Schizophrenia

We used standardized PRS for schizophrenia previously derived by Jones and colleagues^31^ using genome-wide association study (GWAS) data from the second schizophrenia Psychiatric Genomics Consortium (PGC)^37^ and the PLINK score command. PRS were calculated for ALSPAC children by adding the number of risk alleles in each single nucleotide polymorphism (SNP, 0–2) weighted by the logarithm of its odds ratio (OR) for schizophrenia in the PCG sample,^31^ as follows:

$$PRS\;=\sum x_{i}\times \log(OR_{i})$$

In line with previous ALSPAC studies,^31,38^ we used PRS derived using SNPs with a GWAS training-set *P*-value of *P* ≤ .05, because this threshold best captures schizophrenia liability.^37^As null GWAS effects are commonly included in PRS, we further used PRS generated using SNPs meeting a range of *P*-value thresholds (1 × 10^–7^ to 0.5) to investigate sensitivity to a range of cut-offs to denote genetic liability for schizophrenia.^39^ Analyses using PRS data were restricted to the white Caucasian sample because current PRS scores only have sufficient discriminant validity in individuals of European ancestry.^40^ We did not include any covariates accounting for possible population ancestry effects, as previous studies in this sample have shown that there is no significant population stratification, and genome-wide analyses with other phenotypes indicate a low lambda.^41–44^

### Other Variables

In our analyses of the relationship between neighborhood characteristics at birth and symptom outcomes, we included several child and maternal characteristics as potential confounders, including participant ethnicity (white/non-white), maternal marital status (never married/married/widowed, divorced, separated), highest maternal academic education (up to secondary school/university degree or higher), maternal social class (manual/nonmanual occupation), maternal age, and maternal depressive symptoms (total score on the Edinburgh Postnatal Depression Scale^45^). All measures were collected in pregnancy. Participant sex was not included as a confounder, because we hypothesized a priori that child sex would not influence parental residential choice at offspring birth. As sensitivity analyses (as paternal data are characterized by high levels of missing data), we further adjusted our models for paternal age at participant birth, paternal social class (manual/nonmanual profession maternally reported at 32-week gestation) and paternal-reported lifetime history of mental health problems (ie drug addiction, alcoholism, eating disorders, schizophrenia, severe depression, any other psychiatric problem) at 32-week gestation. We also adjusted for self-reported maternal lifetime mental health problems at 12 weeks of gestation (ie drug addiction, alcoholism, eating disorders, schizophrenia, severe depression, any other psychiatric problem).

### Data Analysis

We described the sample with complete exposure information according to covariate and outcome data using cross tabulations with percentages, and means with standard deviations. To test whether children with greater schizophrenia PRS were more likely to be born in more densely populated, deprived, unequal, and socially fragmented areas, we used univariable multinomial logistic regression models, including supplemental analyses to test these associations across various PRS *P*-value thresholds.

To investigate the association between neighborhood characteristics at birth and symptom outcomes we ran 3 logistic regression models: a crude model; a model adjusted for all maternal and child sociodemographic indicators (adjusted model 1), and; a final model mutually adjusted for all neighborhood variables (adjusted model 2). In addition, we reran our final model with and without additional adjustment for PRS for schizophrenia in a sample restricted to participants of white ethnicity.

We used multiple imputation by chained equations to handle missing covariate and outcome data (see supplementary material).^46^ As sensitivity analyses, we (1) additionally adjusted our models for paternal characteristics, as described earlier, and (2) reran our models on participants with complete data only, reporting any notable differences from our primary analyses. All analyses were conducted in Stata 13.^47^

## Results

### Sample

A total of 11 879 children had data available on neighborhood exposures at birth. Of these, 4293 (36.1%) also had data on negative symptoms at age 16 years and 3972 (33.4%) on psychotic experiences at age 18 years. Compared with the rest of England, ALSPAC neighborhoods at birth (ie 1991) were less deprived and socially fragmented, but had similar levels of inequality and were more densely populated (supplementary table S1).^30^ Participants with missing outcome data were more likely to be born in more densely populated and deprived areas, be boys, and be from non-white ethnic backgrounds, with an unmarried, younger mother at child birth, who had only completed secondary education, was from a manual social class and had greater depressive symptoms during pregnancy (supplementary table S2).

Participants of non-white ethnicity, and whose mothers were unmarried, younger at child birth, from a manual social class and had more depressive symptoms during pregnancy were more likely to live in more densely populated, deprived and socially fragmented neighborhoods at birth (table 1). Participants whose mothers had lower educational attainment were more likely to be born in more deprived neighborhoods, but higher maternal education was associated with birth in more densely populated and socially fragmented neighborhoods. Inequality was less clearly associated with other sample characteristics, but lower neighborhood inequality at birth was associated with non-white ethnicity, unmarried mothers, and manual social class.

**Table 1.** Sample Characteristics by Neighborhood Exposure Variables (Based on Participants With Complete Exposure, *N* = 11 879)

	Neighborhood Deprivation				Neighborhood Population Density			
	First Tertile (Least Deprived) *n* (%)	Second Tertile *n* (%)	Third Tertile (Most Deprived) *n* (%)	*P* (*χ*^2^)	First Tertile (Most Rural) *n* (%)	Second Tertile *n* (%)	Third Tertile (Most Urban) *n* (%)	*p (χ* ^*2*^)
Sex								
Male	2042 (33.2)	2057 (33.4)	2056 (33.4 )	.97	2064 (33.5)	2082 (33.8)	2009 (32.7)	.28
Female	1909 (33.4)	1914 (33.4)	1901 (33.2)		1901 (33.2)	1879 (32.8)	1,944 (34.0)	
Ethnicity								
White	3556 (34.6)	3538 (34.4)	3189 (31.0)	<.0001	3516 (34.2)	3,451 (33.6%)	3316 (32.2)	<.0001
Non-White	105 (19.9)	133 (24.0)	317 (57.1)		130 (23.4)	148 (26.7%)	277 (49.9)	
Maternal education								
Secondary	3200 (33.0)	3217 (33.1)	3237 (33.9)	<.0001	3248 (33.4)	3308 (34.1)	3148 (32.4)	<.0001
Degree or above	535 (37.3)	538 (37.5)	362 (25.2)		487 (34.0)	398 (27.7)	550 (38.3)	
Maternal marital status								
Single	364 (16.6)	640 (29.3)	1182 (54.1)	<.0001	578 (26.4)	682 (31.2)	926 (42.4)	<.0001
Married	3328 (38.4)	3041 (35.1)	2291 (26.5)		3067 (35.4)	2925 (33.8)	2668 (30.8)	
Widowed/divorced	188 (27.4)	195 (28.5)	302 (44.1)		215 (31.4)	232 (33.9)	238 (34.7)	
Maternal social class								
Manual	457 (25.4)	579 (32.1)	765 (42.5)	<.0001	562 (31.2)	619 (34.4)	620 (34.4)	.007
Non-manual	2739 (37.8)	2578 (35.7)	1913 (26.5)		2541 (35.1)	2357 (32.6)	2332 (32.3)	
	Mean (*SD*)	Mean (*SD*)	Mean (*SD*)	*P*(*F*)	Mean (*SD*)	Mean (*SD*)	Mean (*SD*)	*P*(*F*)
Maternal Age	29.1 (4.5)	28.4 (4.8)	26.8 (5.1)	<.0001	28.6 (4.9)	27.9 (4.8)	27.8 (5.0)	<.0001
Maternal depressive symptoms	6.5 (4.8)	6.8 (5.0)	7.9 (5.3)	<.0001	6.9 (5.1)	6.9 (5.0)	7.3 (5.2)	.001
Polygenic risk scores^a^	−0.03 (0.9)	−0.02 (1.0)	0.04 (0.9)	.07	−0.02 (1.0)	−0.03 (1.0)	0.02 (0.9)	.2063
	Neighborhood inequality				Neighborhood social fragmentation			
	First tertile (least unequal) *n* (%)	Second tertile *n* (%)	Third tertile (most unequal) *n* (%)	*P* (χ^2^)	First tertile (least fragmented) *n* (%)	Second tertile *n* (%)	Third tertile (most fragmented) *n* (%)	*p* (*χ*^**2**^)
Sex								
Male	2076 (33.7)	2063 (33.5)	2016 (32.8)	.29	2081 (33.8)	2027 (32.9)	2047 (33.3)	.41
Female	1957 (34.2)	1842 (32.2)	1925 (33.6)		1881 (32.8)	1944 (34.0)	1899 (33.2)	
Ethnicity								
White	3453 (33.6)	3364 (32.7)	3466 (33.7)	<.0001	3531 (34.3)	3512 (34.2)	3240 (31.5)	<.0001
Non-White	219 (39.5)	225 (40.5)	111 (20.0)		103 (18.6)	130 (23.4)	322 (58.0)	
Maternal education								
Secondary	3275 (33.8)	3224 (33.2)	3205 (33.0)	.39	3386 (34.9)	3348 (34.5)	2970 (30.6)	<.0001
Degree or above	510 (35.5)	459 (32.0)	466 (32.5)		350 (24.4)	374 (26.1)	711 (49.5)	
Maternal marital status								
Single	820 (37.5)	715 (32.7)	651 (29.8)	<.0001	519 (23.7)	717 (32.8)	950 (43.5)	<.0001
Married	2860 (33.0)	2816 (32.5)	2984 (34.5)		3157 (36.5)	2,902 (33.5)	2601 (30.0)	
Widowed/divorced	240 (35.0)	241 (35.2)	204 (29.8)		193 (28.2)	238 (34.7)	254 (37.1)	
Maternal social class								
Manual	657 (36.5)	575 (31.9)	569 (31.6)	.04	567 (31.5)	688 (38.2)	546 (30.3)	<.0001
Non-manual	2409 (33.3)	2397 (33.2)	2424 (33.5)		2490 (34.4)	2336 (32.3)	2,404 (33.3)	
	Mean (*SD*)	Mean (*SD*)	Mean (*SD*)	*P*(F)	Mean (*SD*)	Mean (*SD*)	Mean (*SD*)	*P*(*F*)
Maternal Age	28.0 (4.9)	28.1 (4.9)	28.2 (4.8)	.12	28.5 (4.8)	27.8 (4.9)	28.0 (5.0)	<.0001
Maternal depressive symptoms	7.0 (5.2)	7.2 (5.1)	7.0 (5.0)	.15	6.7 (5.0)	7.2 (5.1)	7.3 (5.2)	<.0001
Polygenic risk scores^a^	0.004 (0.9)	−0.02 (1.0)	−0.01 (1.0)	.72	−0.07 (0.9)	0.01 (1.0)	0.05 (0.9)	.0007

*Note*: ^**a**^based on participants on white ethnicity only.

### PRS for Schizophrenia and Neighborhood Characteristics

The distribution of PRS for schizophrenia did not differ by neighborhood population density or inequality at birth, but children born in more deprived (third vs first tertile relative risk ratio (RRR): 1.07, 95% CI: 1.01–1.14) and socially fragmented areas (second tertile RRR: 1.07, 95% CI: 1.01–1.14; third tertile RRR: 1.12, 95% CI: 1.06–1.19) had greater PRS for schizophrenia (table 2). The association between PRS for schizophrenia and greater deprivation at birth was consistent across scores derived from SNPs with significance thresholds between 0.05 and 0.5, with weaker evidence at lower thresholds (ie *P* ≤ .01; supplementary figure S1a). The patterns described earlier for social fragmentation, neighborhood inequality, and population density were consistent across all PRS thresholds (supplementary figures S1b and d).

**Table 2.** Association Between Polygenic Risk Score for Schizophrenia and Neighborhood Characteristics at Birth From Multinomial Logistic Regression (*N* = 6887)

	Deprivation	Population Density	Inequality	Social Fragmentation
Tertile	RRR (95% CI)	RRR (95% CI)	RRR (95% CI)	RRR (95% CI)
First (lower)	Ref	Ref	Ref	Ref
Second	1.00 (0.95–1.07); *p* = .82	0.99 (0.93–1.05); *p* = .66	0.98 (0.92–1.04); *p* = .43	1.07 (1.02–1.14); *p* = .02
Third (higher)	1.07 (1.00–1.14); *p* = .03	1.04 (0.98–1.11); *p* = .22	0.99 (0.93–1.05); *p* = .64	1.12 (1.06–1.19); *p* = .0002

*Note*: CI, confidence interval; RRR, relative risk ratio.

### Neighborhood Characteristics and Negative Symptoms

Descriptive statistics suggested there were no overall differences in the prevalence of negative symptoms in adolescence by neighborhood population density, deprivation or inequality at birth (table 3). Greater neighborhood social fragmentation at birth was associated with more negative symptoms in adolescence (*P* = .007). These patterns persisted following multiple imputation in crude and multivariable models (table 3), with children born in the most social fragmentated neighborhoods having greater risk in crude (OR: 1.43, 95% CI: 1.10–1.86), and fully adjusted models (model 2 OR: 1.43, 95% CI: 1.06–1.95); this risk appeared to follow a dose–response pattern. Further adjustment for schizophrenia PRS in the subsample of children of white ethnicity did not alter these findings in our main imputed analyses (table 4), or in complete case analyses (supplementary table S3).

**Table 3.** Association Between Neighborhood Characteristics at Birth and Negative Symptoms at age 16 years, Based on Full Sample With Complete Exposure and Imputed Outcome and Covariates

Exposures	Total N^a^ *N* = 4293	*N* With Exposure and Outcome *n* (%)	*P* (χ^2^) [*P*, χ^2^ for trend]	Negative Symptoms at Age 16 Years (*N* = 11 879)		
				Crude Model OR (95% CI)^b^	Adjusted Model 1 OR (95% CI)^c^	Adjusted Model 2 OR (95% CI)^d^
Population density						
1 (least densely populated)	1578	145 (9.2)	.95 [.82]	Ref	Ref	Ref
2	1314	125 (9.5)		1.01 (0.80–1.27)	1.01 (0.80–1.27)	1.01 (0.80–1.28)
3 (most densely populated)	1401	132 (9.4)		1.05 (0.85–1.30)	1.01 (0.81–1.26)	0.95 (0.76–1.19)
Neighborhood deprivation						
1 (least deprived)	1706	149 (8.7)	.21 [.10]	Ref	Ref	Ref
2	1511	138 (9.1)		1.08 (0.84–1.39)	1.06 (0.82–1.36)	1.00 (0.76–1.31)
3 (most deprived)	1076	115 (10.7)		1.25 (0.97–1.61)§	1.14 (0.88–1.47)	1.00 (0.74–1.35)
Inequality						
1 (least inequality)	1477	146 (9.9)	.57 [.66]	Ref	Ref	Ref
2	1351	118 (8.7)		0.89 (0.69–1.15)	0.89 (0.69–1.15)	0.90 (0.70–1.16)
3 (most inequality)	1465	138 (9.4)		0.95 (0.74–1.22)	0.96 (0.75–1.24)	1.00 (0.78–1.30)
Social Fragmentation^e^						
1 (least fragmented)	1477	118 (8.0)	.03 [.007]	Ref	Ref	Ref
2	1395	129 (9.3)		1.16 (0.87–1.55)	1.13 (0.85–1.52)	1.15 (0.85–1.55)
3 (most fragmented)	1421	155 (10.9)		1.43 (1.10–1.86)**	1.41 (1.07–1.83)*	1.43 (1.06–1.95)*

*Note*: ^a^*N* Refers to participants with complete exposure who also have outcome data.

^b^Crude model.

^c^Adjusted for child’s ethnicity; maternal age, education, marital status, social class, and depression.

^d^Adjusted for all variables in adjusted model 1 all exposures (population density, deprivation, inequality, and social fragmentation) adjusted for each other.

^e^In model 2, there was evidence that social fragmentation provided a better fit to the data when modeled as a continuous categorical variable (OR per tertile: 1.19, 95% CI: 1.01–1.40, *P* = .035). To test this, we compared this model to a more complex model fitted with the categorical term, via Likelihood Ratio Test (*P* = .86) in complete case analyses because LRT cannot be computed in MI models with cluster robust standard errors.

*P ≤ .05, **P ≤ .01, ***P ≤ .0001.

**Table 4.** Association Between Neighborhood Characteristics at Birth and Psychotic Experiences/Negative Symptoms With and Without Adjustment for PRS for Schizophrenia, Using Logistic Regression With Multiple Imputation Restricted to Children of White Ethnicity

	Negative Symptoms at Age 16		Psychotic Experiences at Age 18	
	Adjusted Model 2 OR (95%CI)^a^ *N* = 10 283	Adjusted Model 3 OR (95%CI)^b^ *N* = 10 283	Adjusted Model 2 OR (95% CI)^a^ *N* = 10 283	Adjusted Model 3 OR (95% CI)^b^ *N* = 10 283
Population density				
1 (least densely populated)	Ref	Ref	Ref	Ref
2	1.02 (0.80–1.31)	1.03 (0.81–1.31)	1.27 (0.91–1.76)	1.26 (0.91–1.76)
3 (most densely populated)	0.94 (0.74–1.20)	0.94 (0.74–1.19)	1.59 (1.15–2.21)**	1.59 (1.15–2.21)**
Neighborhood deprivation				
1 (least deprived)	Ref	Ref	Ref	Ref
2	1.00 (0.75 – 1.32)	1.00 (0.76–1.33)	0.89 (0.65–1.23)	0.90 (0.66–1.23)
3 (most deprived)	0.99 (0.73–1.35)	0.98 (0.72–1.34)	0.98 (0.68–1.42)	0.98 (0.68–1.42)
Inequality				
1 (least inequality)	Ref	Ref	Ref	Ref
2	0.90 (0.69–1.17)	0.90 (0.69–1.17)	1.05 (0.80–1.39)	1.06 (0.80–1.39)
3 (most inequality)	0.98 (0.77–1.28)	0.98 (0.76–1.28)	1.10 (0.81–1.48)	1.10 (0.81–1.48)
Social fragmentation				
1 (least fragmented)	Ref	Ref	Ref	Ref
2	1.18 (0.88–1.60)	1.16 (0.86–1.58)	1.28 (0.91–1.81)	1.28 (0.91–1.81)
3 (most fragmented)	1.48 (1.08–2.01)*	1.44 (1.06–1.97)*	1.19 (0.83–1.70)	1.18 (0.83–1.70)

*Note*: ^a^Adjusted for: child’s ethnicity; maternal age, education, marital status, social class, depression + all exposures (population density, deprivation, inequality, and social fragmentation) adjusted for each other.

^b^Adjusted for model 2 covariates + PRS for schizophrenia.

*P ≤ .05, **P ≤ .01, ***P ≤ .0001.

### Neighborhood Characteristics and Psychotic Experiences

Prevalence of psychotic experiences in adolescence increased with greater neighborhood deprivation, population density, and social fragmentation at birth, with evidence of linear trends for all3 exposures (table 5). Following multiple imputation, we observed a crude association between greater population density at birth and the odds of reporting psychotic experiences in adolescence (highest vs lowest tertile OR: 1.77, 95% CI: 1.33–2.35), which persisted after full multivariable adjustment (model 2 OR: 1.57, 95% CI: 1.14–2.17, table 5). These results were not confounded by PRS in our restricted sample (OR: 1.59, 95% CI: 1.15–2.21; table 4). Similar results were observed in sensitivity analyses of complete cases (supplementary table S4). Crude associations between greater deprivation and social fragmentation at birth and psychotic experiences in adolescence were fully confounded by maternal and family factors (adjusted model 1, table 5).

**Table 5.** Association Between Neighborhood Characteristics at Birth and Psychotic Experiences at Age 18 years, Based on Full Sample With Complete Exposure and Imputed Outcome and Covariates

Exposures	Total N^a^ *N* = 3972	*N* with exposure and outcome *n* (%)	*P* (χ^2^) [*P*, χ^2^ for trend]	Psychotic Experiences at Age 18 (*N* = 11 879)		
				Crude Model OR (95% CI)^b^	Adjusted Model 1 OR (95% CI)^c^	Adjusted Model 2 OR (95% CI)^d^
Population density^e^						
1 (least densely populated)	1397	85 (6.1)	.001 [.0001]	Ref	Ref	Ref
2	1238	98 (7.9)		1.31 (0.97–1.78)	1.24 (0.91–1.70)	1.25 (0.91–1.72)
3 (most densely populated)	1337	134 (10.0)		1.77 (1.33–2.35)***	1.56 (1.16–2.09)**	1.57 (1.14–2.17)**
Neighborhood deprivation						
1 (least deprived)	1493	97 (6.5)	<.0001 [.0001]	Ref	Ref	Ref
2	1413	105 (7.4)		1.13 (0.85–1.51)	1.01 (0.75–1.35)	0.89 (0.65–1.21)
3 (most deprived)	1066	115 (10.8)		1.79 (1.33–2.39)***	1.20 (0.87–1.67)	0.98 (0.68–1.42)
Inequality						
1 (least inequality)	1370	110 (8.0)	.91 [.78]	Ref	Ref	Ref
2	1297	106 (8.2)		1.01 (0.79–1.29)	1.03 (0.80–1.32)	1.06 (0.82–1.36)
3 (most inequality)	1305	101 (7.7)		0.97 (0.75–1.26)	1.03 (0.79–1.36)	1.07 (0.80–1.43)
Social fragmentation^e^						
1 (least fragmented)	1357	85 (6.3)	.01 [.01]	Ref	Ref	Ref
2	1341	118 (8.8)		1.45 (1.07–1.97)*	1.31 (0.96–1.79)	1.32 (0.95–1.83)
3 (most fragmented)	1274	114 (9.0)		1.47 (1.11–1.96)**	1.26 (0.92–1.72)	1.19 (0.85–1.67)

*Note*: ^a^*N* Refers to participants with complete exposure who also have outcome data.

^b^Crude model.

^c^Adjusted for: child’s ethnicity; maternal age, education, marital status, social class, and depression.

^d^Adjusted for all variables in adjusted model 1 + all exposures (population density, deprivation, inequality, and social fragmentation) adjusted for each other.

^e^In model 2, there was evidence that population density provided a better fit to the data when modeled as a continuous categorical variable (OR per tertile: 1.30, 95% CI: 1.10–1.55, *P* = .003). To test this, we compared this model to a more complex model fitted with the categorical term, via Likelihood Ratio Test (*P* = .60) in complete case analyses because LRT cannot be computed in MI models with cluster robust standard error.

*P ≤ .05, **P ≤ .01, ***P ≤ .0001.

### Sensitivity Analyses

Patterns of association between social fragmentation and negative symptoms were comparable between complete cases and imputed analyses (supplementary table S3), whereas the association between social fragmentation, population density, and psychotic experiences became stronger in complete case analyses (supplementary table S4). Adjusting for paternal characteristics and parental history of psychiatric problems did not alter our results (data available from authors).

## Discussion

Using a large, longitudinal cohort of adolescents followed since birth, we found that children born in more densely populated neighborhoods were more likely to report psychotic experiences in late adolescence, with some evidence of a dose–response relationship. In our sample, PRS for schizophrenia did not predict birth into more densely populated neighborhoods and therefore did not confound this association. Children born in more socially fragmented neighborhoods were more likely to report adolescent negative symptoms, even after adjusting for PRS for schizophrenia.^31^ Here, deprivation and inequality at birth were not associated with psychotic symptomatology after adjustment for maternal and familial characteristics. PRS for schizophrenia were weakly associated with birth in more deprived neighborhoods.

### Strengths and Limitations

We used a large, well-characterized general population longitudinal cohort linked to small area census indicators of objectively measured neighborhood characteristics at birth. Unlike maternal recall of neighborhood environments—also associated with future psychotic-like phenomena in children^35^—these measures were free from possible recall bias. We used tertiles rather than continuous neighborhood variables to consider possible nonlinear associations with adolescent positive and negative symptoms, given recent reports with respect to psychotic disorder.^48^

We measured neighborhood characteristics at birth for 2 reasons. First, at this time point we had the least amount of missing data, thus increasing sample size. Second, we wanted to exclude the possibility that *intra-generational* social drift—or reverse causation—could account for our findings, ie social drift of an individual during their lifetime. Although it is unlikely that measuring neighborhood characteristics in early childhood would be problematic in this respect, reverse causation would become an increasing concern the later in childhood and adolescence the social environment was measured, if for example families moved to new areas for reasons related to offspring mental health. Sariaslan and colleagues^28^ have observed that the association between deprivation and population density at age 15 years and subsequent schizophrenia risk disappeared in a within-sibling comparison.^15,49^ Although consistent with the hypothesis that associations between urbanicity and schizophrenia could be due to *inter-generational* social drift, ie selection into certain neighborhoods in previous generations, we found little support for this in our study; adjustment for both familial characteristics and—in a subset of participants—child’s PRS for schizophrenia did not alter our findings. This is consistent with a causal association between early life exposure to certain urban environments and psychotic outcomes in adolescence. Nevertheless, we also acknowledge that the current schizophrenia PRS only accounts for 7% of variance on the liability scale of schizophrenia.^37^ This means that current measures of genetic liability could have low specificity in identifying individuals at risk and future studies will be required to fully take into account genetic confounding.

One limitation of our study was cohort attrition between birth and late adolescence; moreover, children with greater PRS for schizophrenia are more likely to be lost to follow-up,^50^ which may have resulted in underestimates of the effect of PRS on our observed associations. Nonetheless, we used multiple imputation to handle missing outcome and covariate data, modeled using a comprehensive set of imputation variables, including PRS data. These results were consistent with, though more conservative than complete case analyses (supplementary tables S3 and S4), underscoring the validity of our findings. The outcomes we used have been previously used in several studies, ensuring comparability of our findings.^5,8,31,35^ We acknowledge that positive and negative symptoms were measured at slightly different points in adolescence (at approximately 18 and 16 years old, respectively). If, for example, early exposure to population density was only associated with psychotic experiences or negative symptoms at 18 years, but not 16 years old, our differential findings with respect to these 2 outcomes might have been an artefact of age-dependent measurement. Nevertheless, we consider this unlikely. In reality, participants in our sample were assessed close to their 16th and 18th birthdays, but with variability around these ages. Furthermore, previous studies of the association between urban birth and later risk of psychotic disorder,^11,15,16^ show this risk is apparent regardless of when the disorder manifests. We were unable to distinguish between suspected and definite psychotic experiences, or investigate specific symptoms (eg hallucinations, delusions) due to low numbers. Finally, the ALSPAC cohort was recruited in a geographically defined catchment in Southwest England, and may not generalize to other settings (supplementary table S1) or birth cohorts.

### Comparison With Previous Studies

Our research extends the evidence base linking neighborhood social adversity to nonclinical psychotic-like phenomena in several ways. First, to the best of our knowledge, this is the first study to investigate these longitudinal associations using environmental measures at birth in relation to psychotic outcomes in middle and late adolescence in a population-based sample. Newbury and colleagues^51^ have recently shown that urbanicity in mid and late childhood was associated with psychotic experiences at age 12 years, independent of deprivation. Our findings with respect to psychotic experiences at age 18 years extend this research, by showing that the association of population density with psychotic experience may begin in early life. Unlike Newbury and colleagues,^51^ we did not observe a direct effect of objectively measured deprivation on psychotic experiences (or, here, negative symptoms) in adolescence. Nevertheless, we have previously shown that maternally related perceptions of neighborhood discord—including noise, rubbish, graffiti antisocial behaviors and fear of crime—during offspring upbringing are associated with adolescent psychotic experiences in the ALSPAC cohort.^35^ Along with the wider literature, which generally supports associations between deprivation and risk of psychotic symptoms and disorder,^52^ these results suggest that both structural (ie economic) and cultural (ie social) determinants may shape risk across the psychosis continuum.

Second, this is the first study to have investigated the role of PRS for schizophrenia on several markers of the social environment at birth, not limited to population density or deprivation. Although there was evidence that PRS for schizophrenia was associated with greater social fragmentation and deprivation at birth, there was no relationship with inequality or population density, and the latter had a strong association with psychotic experiences in late adolescence. Previously, Paksarian and colleagues^22^found that greater PRS were associated with living in the capital city compared with rural areas of Denmark at birth (OR: 1.14; 95% CI: 1.00–1.31) and age 15 years (OR: 1.24; 95% CI: 1.06–1.46). Although we did not find direct evidence of such an association with respect to population density at birth in our sample (RRR: 1.04, 95% CI: 0.98–1.11), our 95% CI overlapped with those found by Paksarian and colleagues.^22^ This suggests that differences in the definition, estimation, categorization and relative values of neighborhood-level factors could affect comparatibility across studies. For example, our neighborhood definitions were based on small area population density estimates, whereas Paksarian and colleagues^22^ used broader measures to approximate overall settlement size. We recommend that future replications of these findings adopt common standards to define and classify population density and other socioenvironmental factors associated with the incidence of schizophrenia and other psychotic disorders.

Our finding that PRS for schizophrenia predicted deprivation and social fragmentation at birth is novel, extending similar evidence with respect to deprivation measured in adolescence.^23^ In our study, the association between PRS and deprivation was restricted to the highest tertile, similar to threshold effects observed between deprivation and the incidence of psychotic disorders.^48,53^ Our results are consistent with evidence for some genetic selection into certain neighborhoods at birth, though in our study this had little impact on later risk of psychotic or negative phenomenology. The association between PRS for schizophrenia and deprivation and social fragmentation at birth may also index individual-level maternal characteristics, such as low income or single marital status, which were also associated with greater PRS in this study and which were included as indicators within these neighborhood-level constructs. Thus, the association between PRS for schizophrenia and deprivation/social fragmentation at birth may summarize a set of genetic and social compositional factors which index individual liability for psychosis.

Finally, the absence of an association between population density at birth and negative symptoms in adolescence is consistent with one cross-sectional study of first episode psychosis,^21^ but contrasts another based on an adult general population sample.^20^ Comparisons with these studies are difficult because of differences in samples study designs and instruments used. We did observe an association between negative symptoms and greater social fragmentation at birth, although the specificity of this finding to psychosis requires consideration; several negative symptom dimensions, including anhedonia, anergia, and avolition might be common to depression.^54^ Although the evidence linking the social environment to depression is more mixed than for psychosis,^55,56^ a recent Dutch cross-sectional study found that neighborhood social characteristics, including low socioeconomic status and the proportion of people on benefits, were associated with rates of depression, but that population density was not.^29^ In the ALSPAC sample, we have also previously shown that low levels of maternally reported neighborhood cohesion and high levels of neighborhood stress in childhood predict offspring depressive symptoms at age 18 years.^35^ Our results, and other,^57^ are compatible with the possibility that early social fragmentation may influence future risk of depression as well as psychosis.

### Meaning of Findings

One interpretation of our findings with respect to population density is that this component of urbanicity exerts a greater effect on positive rather than negative psychotic symptoms, consistent with patterns observed in clinical^21^ and nonclinical samples.^12^ In a previous study in this sample, we have shown that maternal-reported neighborhood characteristics are stable over time.^35^ Hence it is likely that children born in more densely populated neighborhoods would have still resided there later on in their childhood, when exposure to these characteristics might become more salient to the etiology of psychotic experiences. For instance, more densely populated environments may require more frequent and diligent monitoring and processing of social stimuli, and theoretically enhancing stress sensitivity, and resulting in impairments to social cognition—both of which have been associated with psychotic experiences.^6,58^ Urban upbringing has recently been associated with over-activation of the perigenual anterior cingulate cortex (pACC), involved in regulating amygdala activity—a brain region which plays a key role in emotion recognition, stress processing and the so-called “fight or flight” response.^59^ Structural changes in the pACC have also been observed in early stages of schizophrenia,^60^ whereas functional over-activation of this region amongst healthy volunteers experiencing discrimination^61^ suggests that some social exposures encountered in urban environments may disturb neurobiological pathways relevant to psychotic symptoms and disorder. As threat responses are known to involve coordination of dopaminergic signaling across the extended amygdala network,^62^ it is plausible that being born into more urban environments sensitizes this neurobiological pathway, potentially leading to the emergence of aberrant salience of environmental stimuli,^63,64^ as observed in individuals with schizophrenia, and inherently tied to positive symptomology.^58^ Studies integrating genetic information, cognitive measurements, and biomarkers of stress sensitivity in large epidemiological studies of environmental effects on psychosis are required to disentangle how this interplay contributes to risk of psychotic experiences and disorders.

## Funding

This work was supported by a Sir Henry Dale Fellowship to Dr Kirkbride, jointly funded by the Wellcome Trust and the Royal Society (grant no. 101272/Z/13/Z). This work was also supported by the NIHR Biomedical Research Centre at University College London Hospitals NHS Foundation Trust and University College London (UCLH NIHR). Prof Zammit was supported by the NIHR Biomedical Research Centre at the University Hospitals Bristol NHS Foundation Trust and the University of Bristol. This publication is the work of the authors and all authors will serve as guarantors for the contents of this article. The UK Medical Research Council (MRC) and Wellcome (grant ref. 102215/2/13/2) and the University of Bristol provide core support for ALSPAC. A comprehensive list of grants funding is available on the ALSPAC website. The collection of exposure and outcome measures used in this study was funded by the following grants: The Wellcome Trust and MRC (grant no. 076467/Z/05/Z), The Wellcome Trust (grant no. WT091310), and MRC (grant no. MR/M006727/1). Data on the outcome measures used in this study were collected through a grant funded by the MRC (grant no. G0701503/85179) awarded to Prof Zammit. GWAS data were generated by Sample Logistics and Genotyping Facilities at Wellcome Sanger Institute and LabCorp (Laboratory Corporation of America) using support from 23andMe.

## Supplementary Material

sbz049_suppl_Supplementary-Material
