# Supplementary material for: Neighborhood Characteristics at Birth and Positive and Negative Psychotic Symptoms in Adolescence: Findings From the ALSPAC Birth Cohort
Source: Schizophr Bull. 2019 Jun 5;46(3):581–91. doi: 10.1093/schbul/sbz049 (PMC7147568; doi:10.1093/schbul/sbz049)
Supplement: sbz049_suppl_Supplementary-Material [file sbz049_suppl_supplementary-material.docx]

**SUPPLEMENTAL MATERIAL**

**Neighbourhood measures**

We derived measures of neighbourhood-level population density, deprivation, inequality and social fragmentation from 1991 Census data. Population density was estimated as the total usual resident population in each ED divided by its area size (people per km^2^). Neighbourhood deprivation was measured using the Townsend deprivation index, composed of the sum of z-standardised scores of four indicators: percentage of those aged 16 years or over who were economically inactive, percentage of households not owning a car, percentage of non-home ownership, and household crowding. Likewise, social fragmentation was derived from the percentage of single person households, privately rented households, people in households who moved in the last year and unmarried people aged 16 and over. We derived neighbourhood inequality using a Gini-based measure of the dispersion of Townsend deprivation scores between ED within each electoral ward (a higher order of Census geography in which ED are nested), where 0 indicated total equality in deprivation across an electoral ward, and 1 indicated total inequality. These indicators were derived for all ED in England, with relevant values extracted for the ALSPAC sample. From these continuous measures, we derived categorical variables by splitting our sample into equally-sized tertiles, where the third (i.e. highest) tertile indicated people living in the most densely populated, deprived, socially fragmented and unequal areas.

**Multiple imputation**

We imputed 50 datasets using linear, logistic, and multinomial logistic regressions, and constructed imputation models which included several auxiliary variables as well as all variables that we employed in our analyses, as recommended in the literature.48 Auxiliary variables included birthweight, polygenic risk scores for schizophrenia, and a measure of: autistic traits (at age seven years), IQ (at nine years), and depressive symptoms (at age 12 and 18 years). We controlled for the clustered nature of the data (individuals within neighbourhoods) by including the cluster as an indicator in multiple imputation models, and fitting multinomial logistic regressions with cluster-robust standard errors.

**Supplemental figure 1: Flowchart of study participation**

N = 14,062

Core ALSPAC sample

N = 13,998 (99.5%^a^)

Alive at 1 year

N = 11,879 (90.4%^b^)

with geocoded postcode

N = 2,119 (15.1%^b^) with no postcode data or ED data that could not be linked to a census indicator, and twin “B”

N = 64 (0.5%^a^)

(deaths prior to 1 year of age)

^a^= proportion refers to total core ALSPAC sample (N = 14,062)

^b^= proportion refers to N= 13,998

**Supplemental Table 1: Distribution of neighbourhood-level exposures in the ALSPAC sample, by tertile, relative to England**

| **Neighbourhood characteristic** | **ALSPAC participants**  **N (%)** | **Enumeration Districts**  **N (%)** | **ALSPAC^b^**  **Median (IQR)** | **England^b^**  **Median (IQR)** | **Mann-Whitney U p-value** |
| --- | --- | --- | --- | --- | --- |
| Population density (per km^2^) |  |  | 5,480 (2,835 – 7,950) | 4,178 (990 – 7356) | p<0.0001 |
| Low | 3951 | 705 (41.0) | 1,144 (168 – 2,633) | - |  |
| Medium | 3971 | 531 (30.9) | 5,425 (4,620 – 6,200) | - |  |
| High | 3957 | 485 (28.2) | 9,080 (7,863 – 11,167) | - |  |
| Townsend deprivation index^c^ |  |  | -1.44 (-3.26 – 1.03) | -0.66 (-2.76 – 2.41) | p<0.0001 |
| Low | 3965 | 547 (31.8) | -3.74 (-4.30 – -3.21) | - |  |
| Medium | 3961 | 602 (35.0) | -1.45 (-2.03 – -0.74) | - |  |
| High | 3953 | 572 (33.2) | 1.96 (0.96 – 3.67) | - |  |
| Social fragmentation index^c^ |  |  | -0.97 (-2.2 – 1.06) | -0.63 (-1.95 – 1.27) | p=0.0001 |
| Low | 4033 | 560 (32.5) | -2.61 (-3.17 – -2.17) | - |  |
| Medium | 3905 | 586 (34.1) | -0.99 (-1.44 – -0.48) | - |  |
| High | 3941 | 575 (33.4) | 2.60 (1.09 –5.01) | - |  |
| Inequality (Gini coefficient)^d^ |  |  | 0.13 (0.10 – 0.17) | 0.13 (0.10-0.17) | p=0.94 |
| Low | 3962 | 604 (35.1) | 0.10 (0.08 – 0.11) | - |  |
| Medium | 3971 | 584 (33.9) | 0.14 (0.13 –0.15) | - |  |
| High | 3946 | 533 (31.0) | 0.19 (0.18 –0.21) | - |  |

^a^At birth, ALSPAC participants with available geocoded postal data came from N=1,721 enumeration districts [ED] and N=189 electoral wards

^b^Composed of N=101,963 ED and N=8,592 electoral wards with non-zero population counts and all available neighbourhood exposures

^c^Indices based on 4 composite indicators (see Methods), each z-standardised relative to all enumeration districts [ED] in England with a mean of zero and standard deviation of 1. Each indicator is summed to produce a total score for each index

^d^Based on a Gini coefficient, theoretically ranging from 0 (perfect equality) to 1 (perfect inequality) (see Methods) at electoral ward level, measuring variance in deprivation across nested EDs

**Supplemental Table 2: Univariable logistic regression model testing the association between participants’ socio-demographic characteristics and having missing outcome data at age 16 and 18 years of age among those with complete exposure data.**

| **Exposure data** | **Sample with complete exposure**  **N= 11,879** | |
| --- | --- | --- |
|  | Missing negative symptoms data  N = 7,588 (63.9%) | Missing Psychotic experiences data  N = 7,907 (66.6%) |
| **Population density** |  |  |
| 1 (least densely pop) | Ref | Ref |
| 2 | 1.33 (1.21 – 1.46) | 1.20 (1.09 – 1.31) |
| 3 (most densely pop) | 1.20 (1.10 – 1.32) | 1.06 (0.97 – 1.17) |
| **Neighbourhood deprivation** |  |  |
| 1 (least deprived) | Ref | Ref |
| 2 | 1.24(1.13 – 1.35) | 1.10 (1.00 – 1.20) |
| 3 (most deprived) | 2.03 (1.85 – 2.23) | 1.65 (1.50 – 1.81) |
| **Inequality** |  |  |
| 1 (least inequality) | Ref | Ref |
| 2 | 1.09 (1.00 – 1.20) | 1.03 (0.94 – 1.14) |
| 3 (most inequality) | 0.98 (0.89 – 1.07) | 1.04 (0.95 – 1.14) |
| **Social Fragmentation** |  |  |
| 1 (least fragmented) | Ref | Ref |
| 2 | 1.10 (1.00 – 1.20) | 1.03 (0.93 – 1.12) |
| 3 (most fragmented) | 1.06 (0.96 – 1.16) | 1.09 (0.99 – 1.20) |
| **Sex** |  |  |
| Male | Ref | Ref |
| Female | 0.53 (0.49 – 0.57) | 0.63 (0.58 – 0.68) |
| **Ethnicity** |  |  |
| White | Ref | Ref |
| Non-white | 1.56 (1.30 – 1.89) | 1.30 (1.08 – 1.57) |
| **Highest maternal education** |  |  |
| *Secondary* | Ref | Ref |
| *Degree or above* | 0.35 (0.31 – 0.39) | 0.43 (0.49 – 0.39) |
| **Maternal marital status** |  |  |
| *Single* | Ref | Ref |
| *Married* | 0.50 (0.45 – 0.56) | 0.58 (0.52 – 0.64) |
| *Widowed/divorced* | 0.82 (0.68 – 1.00) | 0.88 (0.73 – 1.07) |
| **Social class** |  |  |
| Manual | Ref | Ref |
| Non - manual | 0.55 (0.50 – 0.62) | 0.61 (0.55 – 0.69) |
| **Maternal age** | 0.92 (0.91 – 0.93) | 0.93 (0.92 – 0.94) |
| **Maternal depressive symptoms** | 1.05 (1.04 – 1.06) | 1.03 (1.02 – 1.04) |

**Supplemental figure 1a: Association between the whole polygenic risk score significance range and tertiles of neighbourhood deprivation**

**Supplemental figure 1b: Association between the whole polygenic risk score significance range and tertiles of neighbourhood social fragmentation**

**Supplemental figure 1c: Association between the whole polygenic risk score significance range and tertiles of neighbourhood inequality**

**Supplemental figure 1d: Association between the whole polygenic risk score significance range and tertiles of neighbourhood population density**

**Supplemental Table 3: Association between neighbourhood characteristics at birth and negative symptoms at age 16 years in complete cases samples**

| **Exposures** | **Total N**  **(n = 3,604)** | **N with exposure & outcome**  **n (%)** | **Negative Symptoms at 16** | | | |
| --- | --- | --- | --- | --- | --- | --- |
|  |  |  | **Crude model**  **OR (95%CI)^a^** | **Adjusted model 1**  **OR (95%CI) ^a b^** | **Adjusted model 2**  **OR (95%CI)^a c^** | **Adjusted model 3**  **OR (95%CI)^d^** |
| **Population density** |  |  |  |  |  |  |
| 1 (least densely pop) | 1,328 (36.9%) | 114 (8.6%) | Ref | Ref | Ref | Ref |
| 2 | 1,091 (30.3%) | 101 (9.3%) | 1.09 (0.86 – 1.37) | 1.09 (0.86 – 1.38) | 1.06 (0.84 – 1.34) | 1.06 (0.79 – 1.42) |
| 3 (most densely pop) | 1,185 (32.9%) | 112 (9.5%) | 1.11 (0.84 – 1.47) | 1.10 (0.83 – 1.47) | 1.00 (0.72 – 1.37) | 0.86 (0.64 – 1.16) |
| **Neighbourhood deprivation** |  |  |  |  |  |  |
| 1 (least deprived) | 1,456 (40.4%) | 117 (8.0%) | Ref | Ref | Ref | Ref |
| 2 | 1,300 (36.1%) | 120 (9.2%) | 1.16 (0.92 – 1.48) | 1.16 (0.92 – 1.47) | 1.11 (0.86 – 1.44) | 1.08 (0.80 – 1.45) |
| 3 (most deprived) | 848 (23.5%) | 90 (10.6%) | 1.36 (1.06 – 1.75)* | 1.32 (1.00 – 1.72)* | 1.16 (0.80 – 1.68) | 0.90 (0.59 – 1.36) |
| **Inequality** |  |  |  |  |  |  |
| 1 (least inequality) | 1,228 (34.1%) | 115 (9.4%) | Ref | Ref | Ref | Ref |
| 2 | 1,140 (31.6%) | 95 (8.3%) | 0.88 (0.71 – 1.09) | 0.87 (0.71 – 1.07) | 0.88 (0.71 – 1.10) | 0.87 (0.66 – 1.14) |
| 3 (most inequality) | 1,236 (34.3%) | 117 (9.5%) | - 1. (0.80 – 1.28) | - 1. (0.81 – 1.29) | 1.10 (0.84 – 1.44) | 1.10 (0.80 – 2.61) |
| **Social Fragmentation** |  |  |  |  |  |  |
| 1 (least fragmented) | 1,234 (34.2%) | 93 (7.6%) | Ref | Ref | Ref | Ref |
| 2 | 1,177 (32.7%) | 109 (9.3%) | 1.25 (0.99 – 1.59)* | 1.25 (0.98 – 1.58) | 1.22 (0.95 – 1.56) | 1.34 (0.99 – 1.80)* |
| 3 (most fragmented) | 1,193 (33.1%) | 125 (10.5%) | 1.44 (1.10 – 1.87)** | 1.46 (1.14 – 1.87)** | 1.42 (0.97 – 2.08) | 1.79 (1.22 – 2.61)** |

*p≤0.05, **p≤0.01, ***p≤0.0001.

^a^Based on complete case sample: N=3,604

^b^Adjusted for: child’s ethnicity; maternal age, education, marital status, social class, depression

^c^Adjusted for all variables in Adjusted model 1 + all exposures (population density, deprivation, inequality, and social fragmentation) adjusted for each other

^d^Based on complete case sample restricted to people of white ethnicity: N=2,632; Adjusted for model 2 covariates + PRS for schizophrenia.

**Supplemental Table 4: Association between neighbourhood characteristics at birth and psychotic experiences at age 18 years in complete cases samples**

| **Exposures** | **Total N**  **(n = 3,326)** | **N with exposure & outcome**  **n (%)** | **Psychotic experiences at age 18** | | | |
| --- | --- | --- | --- | --- | --- | --- |
|  |  |  | **Crude model**  **OR (95%CI)^a^** | **Adjusted model 1**  **OR (95%CI) ^a b^** | **Adjusted model 2**  **OR (95%CI)^a c^** | **Adjusted model 3**  **OR (95%CI)^d^** |
| **Population density** |  |  |  |  |  |  |
| 1 (least densely populated) | 1,181 (35.5%) | 62 (5.3%) | Ref | Ref | Ref | Ref |
| 2 | 1,031 (31.0%) | 78 (7.6%) | 1.48 (1.10 – 1.98)* | 1.41 (1.06 – 1.89)* | 1.43 (1.06– 2.92)* | 1.12 (0.80 – 1.57) |
| 3 (most densely populated) | 1,114 (33.5%) | 104 (9.4%) | 1.86 (1.41 – 2.45)*** | 1.71 (1.30 – 2.26)** | 1.72 (1.30 – 2.25)** | 1.60 (1.16 – 2.22)* |
| **Neighbourhood deprivation** |  |  |  |  |  |  |
| 1 (least deprived) | 1,271 (38.2%) | 77 (6.1%) | Ref | Ref | Ref | Ref |
| 2 | 1,222 (36.7%) | 88 (7.2%) | 1.21 (0.91 – 1.59) | 1.14 (0.86 – 1.51) | 0.96 (0.73 – 1.27) | 0.99 (0.73 – 1.34) |
| 3 (most deprived) | 833 (25.1%) | 79 (9.5%) | 1.62 (1.16 – 2.28)** | 1.28 (0.92 – 1.78) | 0.97 (0.70 – 1.34) | 0.97 (0.59 – 1.57) |
| **Inequality** |  |  |  |  |  |  |
| 1 (least inequality) | 1,149 (34.6%) | 80 (7.0%) | Ref | Ref | Ref | Ref |
| 2 | 1,084 (32.6%) | 83 (7.7%) | 1.11 (0.87 – 1.41) | 1.08 (0.85 – 1.38) | 1.11 (0.89 – 1.40) | 1.14 (0.87 – 1.51) |
| 3 (most inequality) | 1,093 (32.9%) | 81 (7.4%) | - 1. 1.07 (0.75 – 1.53) | - 1. 1.10 (0.76 – 1.60) | 1.19 (0.83 – 1.70) | 1.36 (0.92 – 2.02) |
| **Social Fragmentation** |  |  |  |  |  |  |
| 1 (least fragmented) | 1,134 (34.1%) | 63 (5.6%) | Ref | Ref | Ref | Ref |
| 2 | 1,122 (33.7%) | 91 (8.1%) | 1.50 (1.09 – 2.06)* | 1.41 (1.03 – 1.93)* | 1.42 (1.01 – 2.00)* | 1.34 (0.87 – 2.06) |
| 3 (most fragmented) | 1,070 (32.2%) | 90 (8.4%) | 1.56 (1.12 – 2.18)** | 1.43 (1.01 – 2.04)* | 1.36 (0.96 – 1.93) | 1.37 (0.94 – 2.02) |

*p≤0.05, **p≤0.01, ***p≤0.0001.

^a^Based on complete case sample: N=3,326

^b^Adjusted for: child’s ethnicity; maternal age, education, marital status, social class, depression

^c^Adjusted for all variables in Adjusted model 1 ++ all exposures (population density, deprivation, inequality, and social fragmentation) adjusted for each other

^d^Based on complete case sample restricted to people of white ethnicity: N=2,534; Adjusted for model 2 covariates + PRS for schizophrenia
